# Annual of the Universal Medical Sciences. A Yearly Report of the Progress of the General Sanitary Sciences Throughout the World

**Published:** 1888-12

**Authors:** 


*Annual of the Universal Medical Sciences. A Yearly Report of the Progress of the General Sanitary Sciences throughout the World.* Edited by CHARLES E. SAJOUS, M.D., and Seventy Associate Editors. Philadelphia: F. A. DAVIS. 1888. *Five volumes.*

These splendid volumes command our hearty admiration. As an embodiment, annually to be repeated, of all the recent work in all departments of our profession, nothing on so grand a scale has ever yet been attempted. All the best work of all the world has been laid under contribution; and the work has been collated, summarised and written by men who are always competent to handle it, and who are often themselves leading authorities.

The venture is a daring one: we trust it will command the success it deserves. We have read carefully those articles which deal with subjects with which we are familiar, and we find them to be uniformly able, occasionally excellent, and very rarely deficient either in grasp or in fairness. A few obvious criticisms as to the arrangement and classification are deprecated in the preface, and will be remedied in future numbers.

